# Error in Affiliations

**DOI:** 10.1001/jamanetworkopen.2025.19672

**Published:** 2025-06-03

In the Original Investigation titled “Combination of Glucagon-Like Peptide 1 Receptor Agonist and Thiazolidinedione for Mortality and Cardiovascular Outcomes in Patients With Type 2 Diabetes,”^1^ published March 31, 2025, author Jing-Xing Li’s affiliations inappropriately included the School of Medicine, China Medical University, Taichung, Taiwan; and the Graduate Institute of Clinical Laboratory Sciences and Medical Biotechnology, National Taiwan University, Taipei, Taiwan (Dr Li received degrees from these institutions but was not affiliated with them at the time of this study). The correct affiliation for Dr Li is the Department of Internal Medicine, China Medical University Hospital, Taichung, Taiwan. This article has been corrected.^1^
